# S9-4 Retirement as the starting point of active ageing? A mixed-methods study to identify barriers and facilitators of physical activity when retiring

**DOI:** 10.1093/eurpub/ckad133.046

**Published:** 2023-09-11

**Authors:** Nina Vansweevelt, Filip Boen, Jannique van Uffelen, Jan Seghers

**Affiliations:** KU Leuven; KU Leuven; KU Leuven; KU Leuven

## Abstract

**Purpose:**

The transition from work to retirement implies changes in daily activities and might constitute an opportunity to increase physical activity (PA). While qualitative studies reported that retirees mainly perceived increases in PA during this life transition, quantitative studies observed decreases in total PA. Moreover, changes in total PA seem to depend on socio-economic status, with mostly lower socio-economic groups decreasing their total PA. Therefore, the goal of this study is (1) to investigate subjective changes in PA during the retirement transition and the perceived determinants of these changes, including barriers and facilitators to increasing or maintaining PA; (2) to link this subjective information to device-based measurements of PA.

**Methods:**

This study is part of a mixed-methods study with a parallel structure using quantitative and qualitative methods. In the quantitative, longitudinal part of this study (n = 93), PA is measured with accelerometers prior to retirement, and at three, six and twelve months post-retirement. In addition, one-on-one, semi-structured interviews are being conducted with a subsample six months after retirement (n = 30, stratified on gender and education, randomly selected from the total sample). Thematic analysis is used to analyse the interview data with an inductive approach. The emerged themes will be mapped on the COM-B (Capability, Opportunity, Motivation and Behaviour) and TDF (Theoretical Domains Framework) models.

**Results:**

The data collection is ongoing and more than half of the interviews have been conducted. Based on preliminary analyses of the first 10 interviews, it seems that most participants perceive to have more PA post-retirement, and that time is a main facilitator. Those participants who report having become less physically active were never interested in PA and/or did not value it. Moreover, some participants report having other competing activities, including grandchild-care or sedentary leisure activities. Further analyses will provide more specific conclusions and link qualitative and quantitative results.

**Conclusions:**

The experience of retiring is highly individual and interventions should be personalized to specific target groups. This study will provide useful information on potential keys to develop effective lifestyle interventions for adults during or near the retirement transition.

